# Neuroendocrine Carcinoma of the Gallbladder

**DOI:** 10.7759/cureus.27022

**Published:** 2022-07-19

**Authors:** Daniel A Vidal Panduro, Elizabeth Zegarra Buitron, Omar J Cochella Tizon, Domingo A Morales Luna

**Affiliations:** 1 Internal Medicine, School of Medicine, Universidad Peruana de Ciencias Aplicadas, Lima, PER; 2 Pathology, Hospital Nacional Hipólito Unanue, Lima, PER

**Keywords:** gallbladder neoplasms, gallbladder neuroendocrine carcinoma, neuroendocrine carcinoma, neuroendocrine tumor, gallbladder

## Abstract

Neuroendocrine carcinoma of the gallbladder (NECGB) is a rare, aggressive tumor with a poor prognosis. There are two main categories, well-differentiated NECGB and poorly differentiated NECGB, the latter with a worse prognosis. The clinical presentation is non-specific, but the occurrence is more frequent in women with cholelithiasis. Histologic and immuno-histochemical confirmation is required to establish the diagnosis. Treatment is primarily surgery with or without adjuvant chemotherapy. We present the case of a 43-year-old woman with pain in the right upper quadrant, diagnosed with NECGB following cholecystectomy. Subsequently, she received cycles of chemotherapy.

## Introduction

Neuroendocrine neoplasms (NENs) are tumors that originate from neuroendocrine cells and are present in the digestive, respiratory, and thyroid tracts [1]. Neuroendocrine carcinoma of the gallbladder (NECGB) is a rare and aggressive tumor, accounting for 2.3% of all gallbladder tumors [2]. They occur more frequently in elderly women [3]. The diagnosis is frequently made after cholecystectomy in a patient with a history of cholelithiasis [4]. The main treatment is surgery and depending on the stage of the tumor, adjuvant chemotherapy is recommended [5,6]. Despite aggressive treatment, this type of tumor has a poor prognosis, since it is usually diagnosed in advanced stages with distant metastasis [7]. We present a case of a 43-year-old woman, who underwent cholecystectomy for acute cholecystitis, in whom the diagnosis of NECGB was made. After surgery, she received chemotherapy and currently, she is in follow-up.

## Case presentation

A 43-year-old woman presented to La Oroya Hospital (highlands of Peru) with a one-month history of progressive right hypochondrial and epigastric pain, she was diagnosed as having acute cholecystitis and a laparoscopic cholecystectomy was performed.

**Figure 1:**
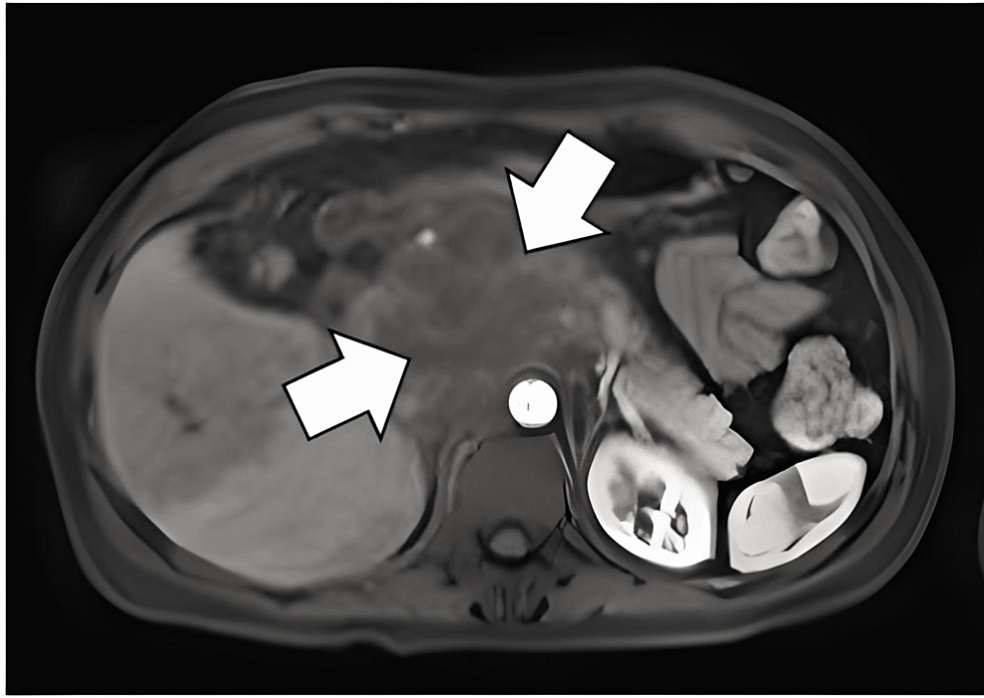
Abdominal magnetic resonance cholangiopancreatography (MRCP) showed the presence of a 47 x 30 x 45 mm lesion at the level of the head and body of the pancreas towards the posterior region with poorly defined irregular borders. Suggestive image of neoproliferative process in the head and body of the pancreas.

Three days later, she presented with abdominal pain in the epigastrium associated with hyporexia and nausea, receiving symptomatic treatment. One day before admission, the symptoms persisted and she went to a private physician where a magnetic resonance cholangiopancreatography (MRCP) was performed; signs of a neoformative lesion appeared in the head and body of the pancreas (Figure 1). For this reason, she was transferred to a tertiary-level hospital in Lima.

**Figure 2:**
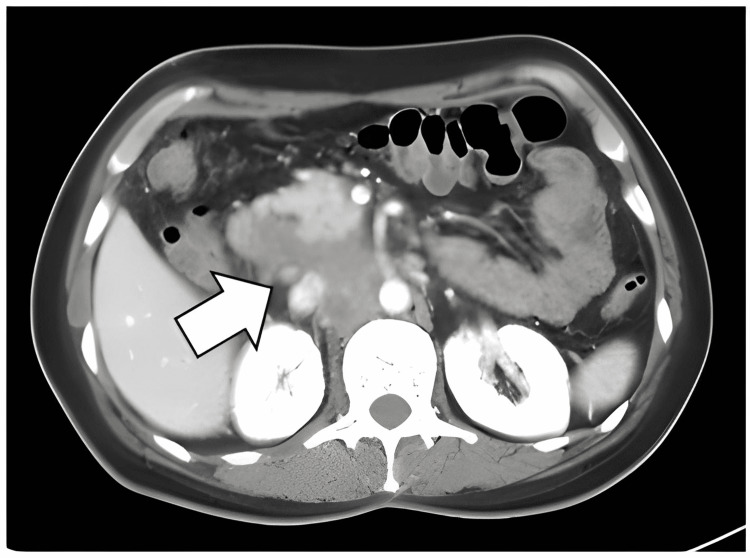
Abdominal CT-scan showed an alteration of pancreatic density with increased cephalic segment volume that included hypodense areas of necrotic aspect that contacted the duodenal wall.

Clinical findings included dry mucous membranes, hypoactive bowel sounds, slight pain in the epigastrium, and a recent operative scar; rest of the examination was non-contributory. She presented with moderate anemia (hemoglobin of 8.3 g/dL), lipase 273 U/L (normal range 0 - 160 U/L), amylase 311 U/L (normal range 40 - 140 U/L), and a PCR of 3.7 mg/L (normal range 0.01 - 5 mg/L); a CT scan was performed which showed alteration of pancreatic density with increased cephalic segment volume, with necrotic-looking areas (Figure 2).

Due to the clinical findings, it was considered a presumptive diagnosis of acute necrotic collections versus malignant neoplasm of the pancreas. On the seventh day of hospitalization, neuron-specific enolase was evaluated and showed a value of 164 ng/mL (normal range < 16.3 ng/mL). Subsequent to this, three days later, total enteral nutrition (TEN) was initiated. Ancillary exams on the same day showed an amylase of 365 U/L, lipase of 353 U/L, lactate dehydrogenase (LDH) of 809 IU/L (normal range 105 - 333 IU/L), and alkaline phosphatase of 133 IU/L (normal range 44 - 147 IU/L); the pain did not decrease by use of analgesics.

**Figure 3:**
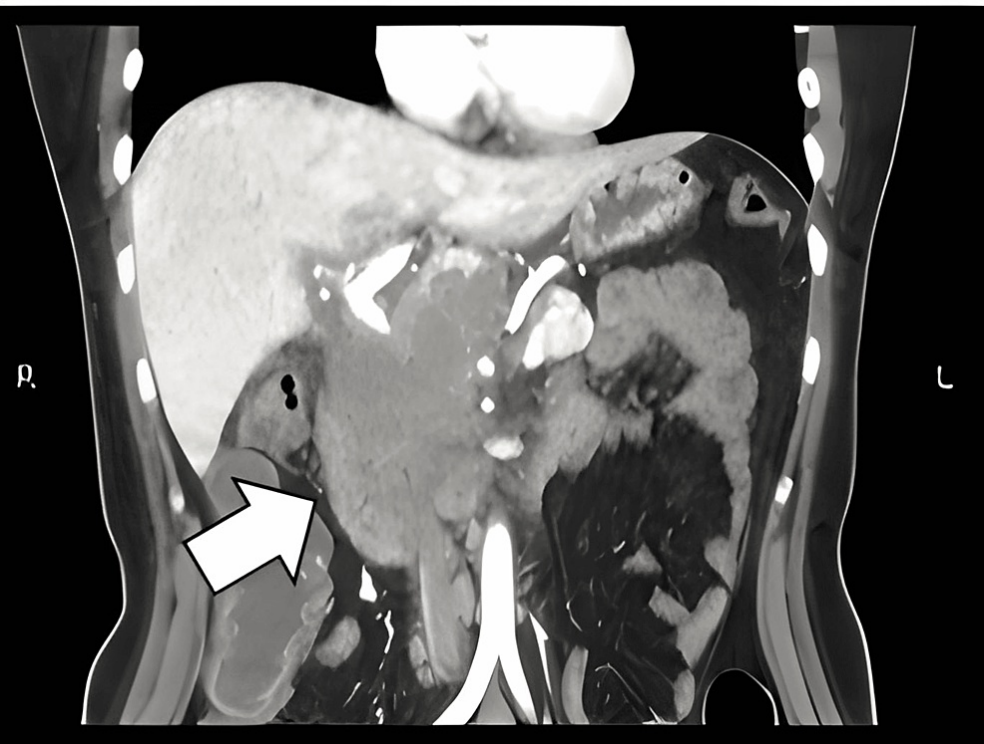
Multislice computed tomography (MSCT) of the pancreas showed adenopathic conglomerate with areas of central degeneration that reached up to 5 cm in a retroperitoneal, peripancreatic, cephalic, retroduodenal, and peri-aortic location. The largest volume was retroperitoneal which conditioned the anterior displacement of the pancreas.

Two days after starting TEN, an LDH increase of 809 IU/L to 2251 IU/L was observed. A multislice computed tomography (MSCT) of the pancreas was performed showing multiple retroperitoneal, retroduodenal, periaortic, and peripancreatic adenopathies (Figure 3).

**Figure 4:**
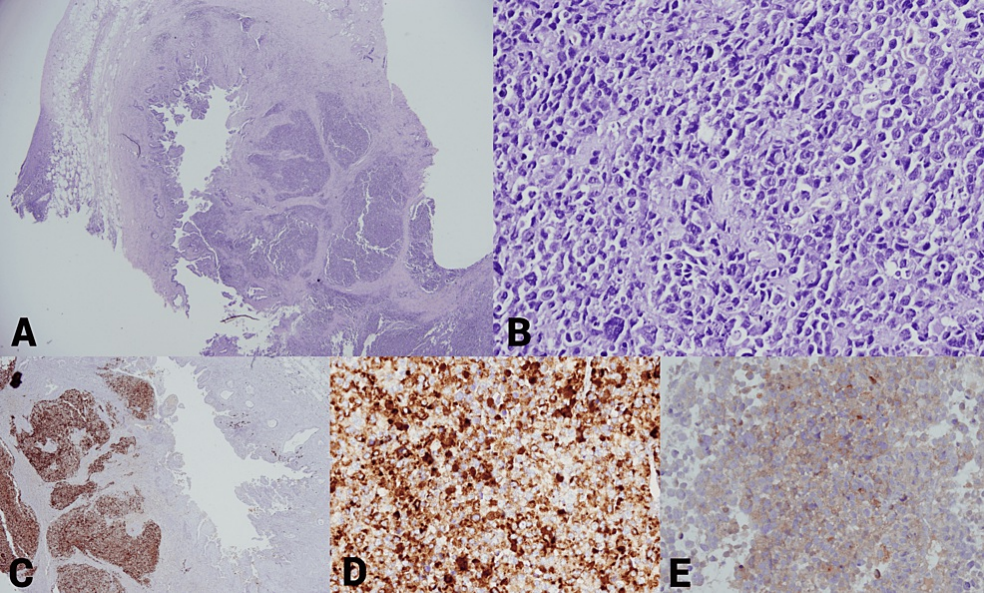
A) Hematoxylin and eosin (H&E) 4x; B) H&E 40x; C) chromogranin A positive 4x; D) chromogranin A positive 40x (cytoplasmic granular staining); E) positive expression of synaptophysin 40x.

An endoscopy was performed, but it was not possible to transpose the pylorus by marked angulation. An echoendoscopy was performed in which a peri pancreatic conglomerate was observed. Fine-needle aspiration (FNA) was also performed and the sample was sent to pathology. Subsequently, the operative report of the laparoscopic cholecystectomy performed in La Oroya was requested. The report showed gallstones with thickened walls with areas of tumor and irregular borders adhering firmly to the liver and hypertrophic sentinel node with hard consistency. The immuno-histochemical analysis showed strong chromogranin A immunoreactivity and positive expression of synaptophysin. Pathological anatomy revealed consistent findings with NECGB grade III (Figure 4).

The patient received combination chemotherapy with cisplatin and etoposide given intravenously. Currently, the patient is under follow-up.

## Discussion

Previously, this type of tumor was called carcinoid tumor [8]. However, it was not until 1995 that the term was changed to neuroendocrine tumor (NET) [9]. NENs are rare and can occur predominantly in the gastrointestinal and respiratory tracts, 66% and 31%, respectively [1], and their incidence is 5.25/100,000 [5]. NECGB accounts for 2.3% of all gallbladder tumors [2] and less than 0.5% of all neuroendocrine carcinomas of the gastrointestinal tract [2,6,7]. The average age of diagnosis is 64 years, with a greater predominance in the female sex, ratio 1.9:1 [3].

According to the World Health Organization (WHO) 2010, NENs of the gastrointestinal tract are classified according to the rate of tumor proliferation, using mitotic counts or the ki-67 marker index [3,10]. There are two main categories of NENs of the gastrointestinal tract: well-differentiated NETs, which include low-grade (G1) and medium-grade (G2); and poorly differentiated neuroendocrine carcinomas (NECs) with mitotic figures >20/10 HPF and Ki-67 staining >20% positive, which includes high-grade carcinoma (G3), small cells and long cells [3,8,11].

There is no presence of neuroendocrine cells in the mucosa of the gallbladder, which is why experts propose different theories of the origin of these tumors [3,12,13]. One of them refers to chronic inflammation caused by cholelithiasis or chronic cholecystitis, which could induce intestinal and/or gastric metaplasia of the gallbladder, resulting in dysplasia and malignant transformation [12,13]. Not all neuroendocrine carcinomas are accompanied by cholelithiasis, but 74%-92% of patients with gallbladder carcinoma and the majority of patients with neuroendocrine carcinoma present with cholelithiasis [4].

According to Matsumo et al, chronic bile reflux of pancreatic secretions can produce metaplasia [14]. On the other hand, neuroendocrine carcinoma can also present concomitantly with an adenocarcinoma and transform from it [15]. In our case, the patient presented acute cholecystitis, which is why she underwent surgery and was subsequently diagnosed with NECGB.

Clinical symptoms at the time of presentation are nonspecific [5,6,11,12], with the most frequent symptom being pain in the right upper quadrant, indistinguishable from cholelithiasis, followed by abdominal discomfort, nausea, vomiting, jaundice, weight loss, and positive Murphy's sign [5,6,8,11,12]. The symptoms of carcinoid syndrome are not frequently reported, since most of these tumors are non-functioning [16]. However, if substances such as histamine and serotonin are not fully degraded, they can cause symptoms such as diarrhea, edema, wheezing, and facial flushing [8,11]. According to Ghosh et al, the most frequent location of metastasis is the liver, followed by abdominal lymph nodes, lungs, and bone [17].

This type of tumor is usually diagnosed after cholecystectomy in patients with cholelithiasis because it is very difficult to differentiate it from other types of gallbladder carcinomas through imaging [3,6,11,12]. The radiological findings that have been described are the presence of a mass that replaces the gallbladder, local or diffuse thickening of the wall, with or without hepatic invasion, hepatic metastatic nodules, and locoregional lymphadenopathy [16]. However, the findings of well-defined margins, liver metastases, and lymphadenopathies are useful to differentiate this type of tumor from adenocarcinoma using tomography [16,18].

The definitive diagnosis, stage, and grade of the tumor are based on the results of the pathological and immunohistochemical analysis, positive for biomarkers such as chromogranin A and synaptophysin, with 91.9% and 84.8% [5,11]. In our case, these two biomarkers presented immunoreactivity. Other markers, also present, are CD56 and Ki67 [19]. It should be borne in mind that when evaluating an advanced gallbladder tumor with tumor markers at normal levels, such as CEA and CA 19-9, the diagnostic possibility of neuroendocrine neoplasia must be considered [3].

Until now, there is no consensus on the definitive treatment of this neoplasm, but the therapeutic strategy depends on the stage of the tumor [5]. For patients with early-stage tumors, cholecystectomy alone is usually sufficient [5], but in patients with more advanced stages, more extensive surgeries with resection of nodules or metastatic lesions are required, followed by courses of chemotherapy [8]. The most recommended therapy is cisplatin or carboplatin plus etoposide [5,6,12]. Carrera et al. reported that the use of cisplatin with etoposide improved four-month survival compared to other chemotherapy regimens [20]. In the case presented, the patient received the same chemotherapeutic regimen. The role of radiotherapy is still uncertain since NETs are generally insensitive to it [3,12].

Since most of these tumors are diagnosed in advanced clinical stages, they have high mortality rates and poor prognosis [7]. Some of these poor prognostic factors are distant metastasis, tumor size greater than 25 mm, long cell neuroendocrine carcinoma, tumor stage 3, and high Ki67 index (>20%) [3,5,6]. Despite treatment, survival is four to six months, with a worse prognosis than gallbladder adenocarcinoma [3,6]. In a study that analyzed 72 cases, the median survival was 13 months, with metastatic lesions in 72% of cases [20].

## Conclusions

NECGB is a rare type of tumor, with a poor prognosis and high mortality, usually detected in advanced stages. Pathological and immuno-histochemical analysis are essential to make a correct and timely diagnosis. It is important to obtain more information on these type of tumors, to establish a definitive consensus on diagnosis and treatment, in order to improve the prognosis and survival of these patients.
